# Survival Analysis and Prediction Model of ASCP Based on SEER Database

**DOI:** 10.3389/fonc.2022.909257

**Published:** 2022-06-24

**Authors:** Sun-Yuan Lv, Min-Jie Lin, Zhao-Qun Yang, Chen-Nan Xu, Zhi-Ming Wu

**Affiliations:** China Medical University, Shaoxing Hospital, Shaoxing, China

**Keywords:** ascp, PSM, SEER (Surveillance Epidemiology and End Results) database, prognosis, nomogram, APC

## Abstract

**Background:**

This study aims to compare the incidence and clinical and survival characteristics of adenosquamous carcinoma of the pancreas (ASCP) and adenomatous carcinoma of the pancreas (ACP), analyze the survival factors of ASCP and construct a prognostic model.

**Method:**

Patients diagnosed with pancreatic cancer from 2000 to 2018 are selected from the SEER database. ASCP and ACP are compared in terms of epidemiology, clinical characteristics and prognosis. Cases are matched in a 1:2 ratio, and survival analysis is performed. The Cox proportional hazard model is used to determine covariates related to overall survival (OS), and an ASCP prognosis nomogram is constructed and verified by consistency index (C-index), calibration chart and decision curve analysis (DCA). The accuracy of the model is compared with that of AJCC.Stage and SEER.Stage to obtain the area under the receiver operating characteristic (ROC) curve.

**Results:**

the age-adjusted incidence of ACP increased significantly over time from 2000 to 2008 and from 2008 to 2018 (P < 0.05). APC was 2.01% (95% CI: 1.95–2.21) and 1.08% (95% CI: 0.93–1.25) respectively. The age-adjusted incidence of ASCP increased with time from 2000 to 2018 (P < 0.05) and APC was 3.64% (95% CI: 3.25–4.01).After propensity score matching (PSM), the OS and cancer-specific survival (CSS) of ACP are better than those of ASCP. The survival time of ASCP is significantly improved by the combined treatment of surgery + chemotherapy + radiotherapy, with a median OS of 31 months. Cox proportional hazard regression analysis shows that age, race, surgery, radiotherapy, chemotherapy and tumor size are independent factors affecting the prognosis. DCA and area under the curve (AUC) value shows that the model has good discrimination ability.

**Conclusion:**

The OS prognosis of ASCP is worse than that of ACP, and the nomogram has high accuracy for the prognosis prediction of ASCP.

## Introduction

Pancreatic cancer is a highly malignant tumor and the deadliest gastrointestinal (GI) cancer, with morbidity and mortality approaching 1 (1). With half a million new cases diagnosed each year worldwide, it is one of the few cancers of which the incidence is still rising in the United States (2, 3). Most pancreatic cancers are adenomatous carcinoma of the pancreas (ACP), and only 0.4–4% are adenosquamous carcinoma of the pancreas (ASCP) (4). Histologically, ASCP is defined as consisting of at least 30% malignant squamous cell carcinoma with coexisting ductal adenocarcinoma (5, 6). Due to its low incidence rate, ASCP has been reported in individual or small cases in most literature (5, 7–12). Despite current advances in surgical techniques and clinical drugs, the overall survival (OS) is less than 1 year (13), with a median OS(MOS) of 12 months for resectable disease and 4–5 months for metastatic disease (14). Compared with ACP, ASCP is more aggressive and potentially metastatic with a worse prognosis (15). However, according to several studies, the OS of ASCP and ACP are inconsistent (8, 16). ASCP is a solid cancer and its prognostic factors are difficult to predict. Currently, the TNM staging system of the American Joint Committee on Cancer (AJCC) is used to evaluate the survival and prognosis of pancreatic cancer patients. Based on tumor size and extent, number of lymph node metastases and number of distant metastases (17, 18), the system provides a simple but incomplete tool for assessing ASCP development and disease treatment and decision-making. According to the reported literature, the independent prognostic factors of ASCP include age, gender, race, radiotherapy, chemotherapy, surgery, anatomic site, etc. (12, 14, 16, 19). Compared with TNM staging, the above factors have a convincing ability to predict the prognosis of ASCP. Thus, further studies on ASCP are necessary.

Surveillance, Epidemiology and End Results (SEER) (https://seer.cancer.gov/) is the largest and most authoritative database of tumor-related information in the United States, which collects tumor incidence and survival data from population-based cancer registries covering approximately 34.6% of the U.S. population (20). Studies combined with the SEER database can be targeted at different regions and larger populations, and population analysis can produce more convincing results for the rare tumor ASCP. Big data can generate individual probabilities of clinical events, meeting our needs for integrated biological and clinical models (21, 22). Therefore, this study explores the epidemiology, clinical characteristics and prognosis of ASCP and ACP through the SEER database. In addition, we analyzed the prognostic risk factors for ASCP and constructed a nomogram to provide clinicians with a convenient tool for implementing individualized treatment.

## Materials and Methods

### Ethical Approval

Informed consent is not required for SEER data, and this study is consistent with the 1964 “Declaration of Helsinki” and its subsequent amendments or similar ethical standards.

### Patients

The data for this study was obtained from the SEER database, covering up to 97% of cancer incidence and 28% of the U.S. population, and obtained by SEER*Stat v8.3.9 under the registration number 17070-Nov2020.

Inclusion criteria: 1. International Classification of Diseases for Oncology ICD-0-3/WHO 2008 = Pancreas; 2. patients diagnosed between 2000 and 2018; 3. histologically, ACP under ICD-0-3 His/behav = 8140, 8141, 8142, 8144, 8500, 8501, 8503, 8504, 8507 and 8521, and ASCP under ICD-0-3 His/behav = 8560. Exclusion criteria: 1. previous malignant tumors and multiple tumors; 2. non-pathologically/cytologically confirmed patients and autopsy results; 3. unknown surgery information; 4. tumor size = 0 cm; 5. T0 Stage; 6. unknown survival time. OS is defined as the interval between cancer diagnosis and the last follow-up of patients who have died by any cause or are still alive. Cancer-specific survival (CSS) is defined as the time interval between cancer diagnosis and death from pancreatic cancer.

### Statistical Analysis

Demographic and clinical characteristics were extracted from the SEER database, including age, gender, race, grade, tumor site, SEER stage, AJCC stage, T stage, lymph node metastasis, distant metastasis, adjuvant therapy (chemotherapy and radiation therapy) surgery and vital conditions at follow-up. Age-adjusted incidence per 100,000 patients diagnosed per year was calculated based on the number of cases. Annual percentage change (APC) in incidence change was assessed using Joinpoint software, and random 1:2 nearest neighbor propensity score matching (PSM) was used to balance all baseline covariates between ASCP and ACP. ASCP cases in the SEER database were then randomly assigned to the training and validation groups in a ratio of 7:3. The classified data was expressed as frequency and percentage, and verified by the Chi-square test or Fisher’s exact test. Continuous data was expressed as median and standard deviation (SD), and compared by the Mann-Whitney U test. The survival curvess were plotted using the Kaplan-Meier method and compared by the log series test. The Cox proportional hazard regression model was used for univariate and multivariate analyses, and the hazard ratio (HR) and corresponding 95% confidence interval (CI) were calculated. P values greater than 0.05 on both sides were considered statistically significant. All statistical analyses were performed using R-version 4.1.2 (R Foundation for Statistical Computing, Vienna, Austria, http://www.r-project.org) and related software packages. In the training group, a probability nomogram of OS at 6, 12 and 24 months was constructed on the basis of independent prognostic risk factors screened by Cox proportional hazard regression, then compared using AJCC.Stage and SEER.Stage to calculate the nomogram score. The sensitivity of the nomogram was assessed by C-index. A calibration chart (1,000 bootstrap resamples) was used to evaluate the consistency between model prediction and actual prediction, and DCA was used to evaluate the clinical effects and clinical benefit capacity of the nomogram. Finally, the accuracy of the nomogram, AJCC.Stage and SEER.Stage was compared using the area under the ROC curve.

## Results

### Incidence of ACP and ASCP

**Figure 1:**
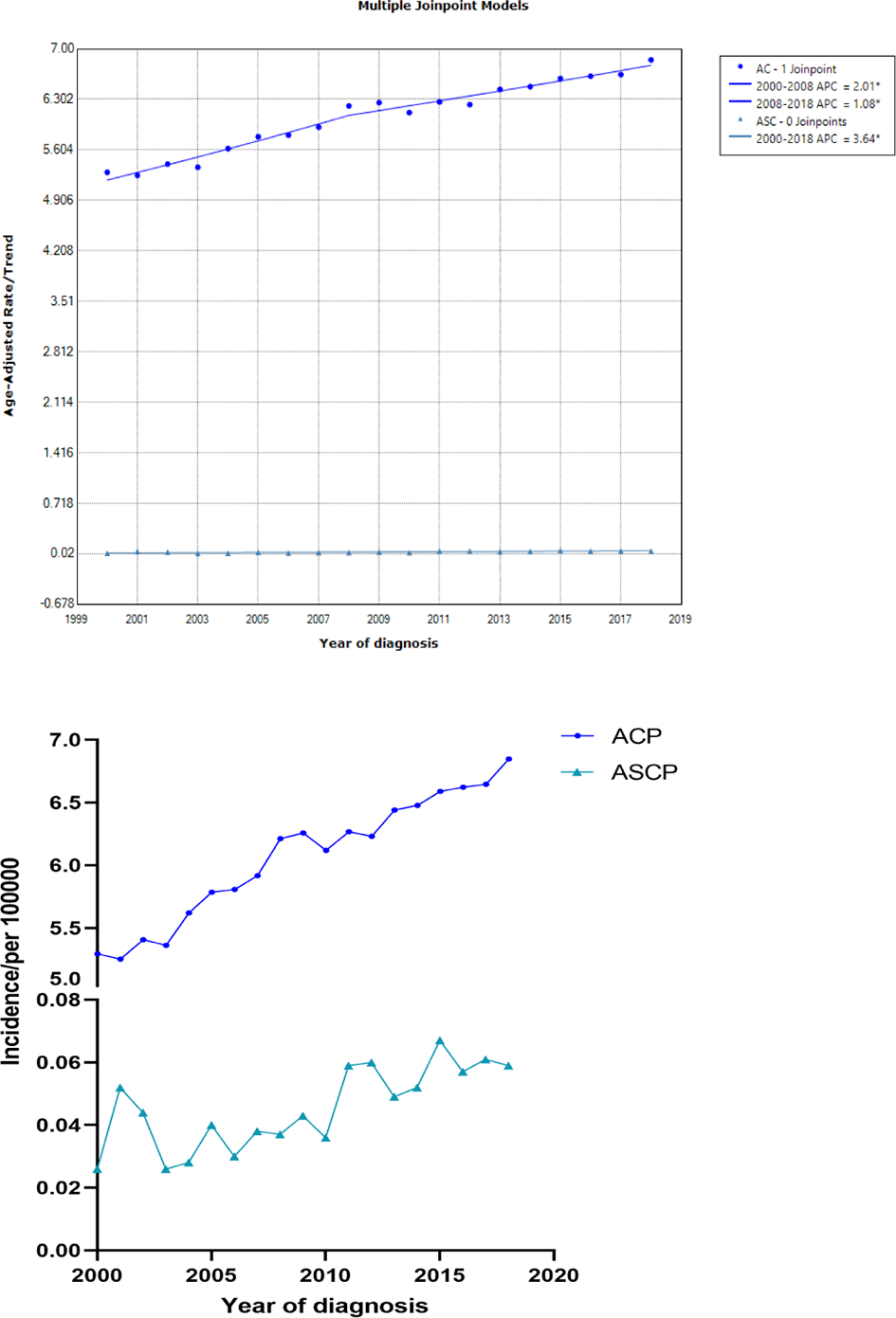
The age-adjusted incidence of ACP and ASCP patients between 2000 and 2018 from the SEER database(ACP, Pancreatic adenocarcinoma; ASCP, Pancreatic adenosquamous carcinoma; APC, annual percent change).

In this study, patients with ASCP and ACP from 2000 to 2018 were selected from the SEER database, and the incidence trend was described using Joinpoint software. As shown in **Figure 1**, the age-adjusted incidence of ACP increased significantly over time from 2000 to 2008 and from 2008 to 2018 (P < 0.05). APC was 2.01% (95% CI: 1.95–2.21) and 1.08% (95% CI: 0.93–1.25) respectively, but the incidence of pancreatic adenocarcinoma slowed slightly with time after 2008. The age-adjusted incidence of ASCP increased with time from 2000 to 2018 (P < 0.05) and APC was 3.64% (95% CI: 3.25–4.01). The detailed incidence data of ACP and ASCP is shown in **Supplementary Table 1** (2000–2018). (**Supplementary Figure 1** shows the age-adjusted incidence figures of ASCP and ACP from 1975 to 1999, and **Supplementary Table 2** shows the detailed incidence data from 1975 to 1999).

### Demographic and Clinical Characteristics

**Figure 2:**
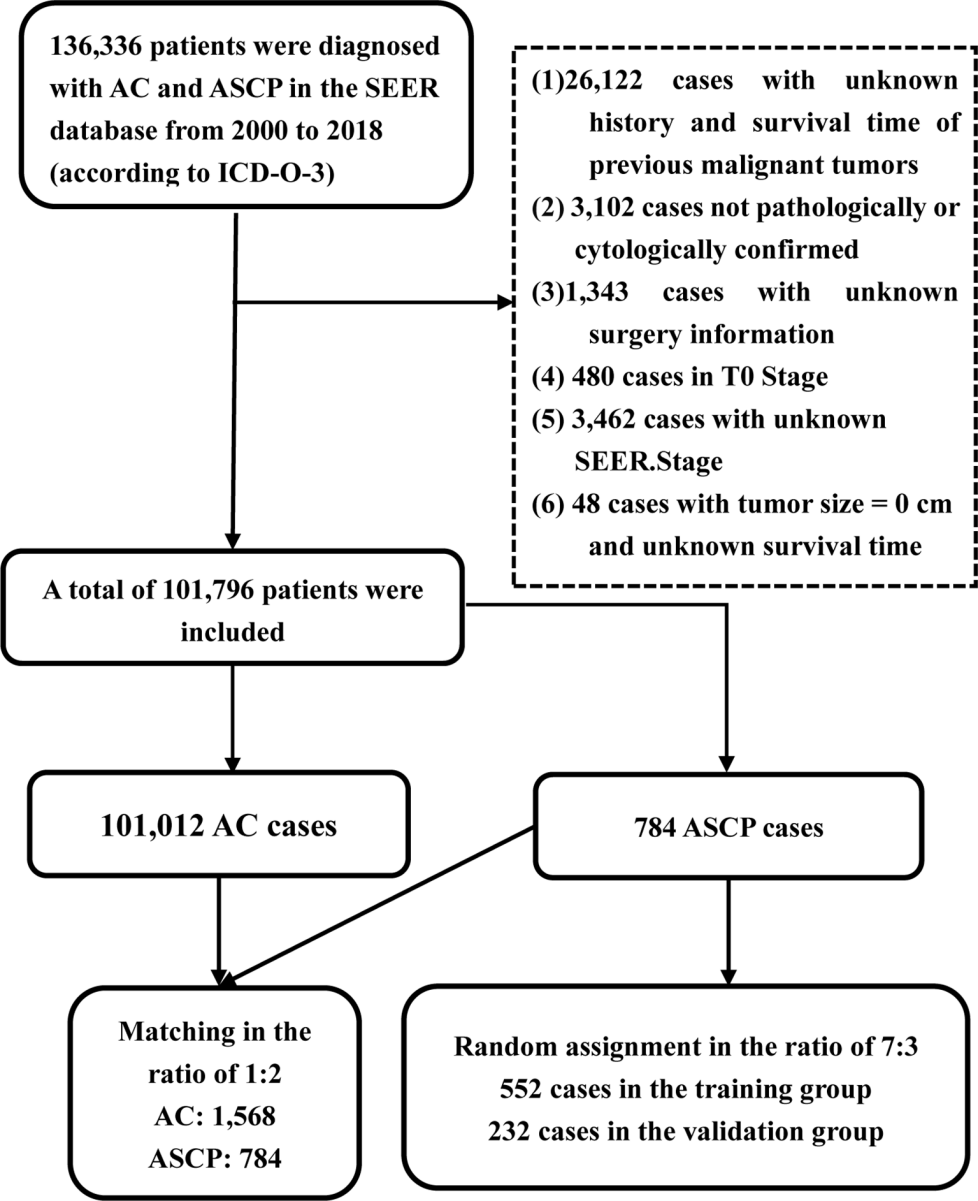
Flowchart for Selecting Patients from SEER Database.

From 2000 to 2018, a total of 136,336 patients in the SEER database (according to ICD-O-3) were diagnosed as having ACP and ASCP. According to the inclusion and exclusion criteria, 101,796 patients were finally included in this study, including 101,012 ACP cases and 784 ASCP cases (**Figure 2**). The clinical characteristics of two different tumor subtypes of two different pancreatic cancers are summarized in **Table 1**. It can be seen that in terms of demographic characteristics, the average age at diagnosis in both groups was around 67 years old, and the incidence was slightly higher in men than women, accounting for 51.9% in ACP and 52.3% in ASCP. There was a significant increase in the proportion of white patients compared with that of other races at 79.2% and 81.0% respectively. In terms of clinical characteristics, the incidence of cancer of the head of the pancreas was higher than that of other parts, accounting for 52.4% in ACP and 44.6% in ASCP. Interestingly, distant metastasis was higher in ACP than in ASCP at 44.6% and 37.9% respectively. the proportion of patients undergoing surgery in ASCP is higher than that of ACP (39.4% vs. 21%), and the proportion of patients with ASCP who received chemotherapy and radiotherapy was similar to that of ACP patients. To eliminate differences in baseline characteristics between the two groups, PSM was used to balance all characteristics, including age, gender, race, grade, tumor site, SEER.Stage, AJCC.Stage, T stage, lymph node metastasis, distant metastasis, radiotherapy, chemotherapy and surgery. After PSM, there were no significant differences in demographic and clinical characteristics between the two groups of patients (P > 0.05).

**Table 1:** Comparison of clinicopathological characteristics between ASCP and ACP.

Characteristic	Before PSM		P	After PSM		P
	ACPn=101012 (%)	ASCPn= 784 (%)		ACPn=1568 (%)	ASCPn=784(%)	
**Age**	67.4(11.4)	67.9(10.7)	0.171	67.5 (10.6)	67.9 (10.7)	0.335
**Race:**			0.312			0.561
White	80051 (79.2%)	635 (81.0%)		157(10.0%)	84 (10.7%)	
Black	12646 (12.5%)	84 (10.7%)		114 (7.27%)	65 (8.29%)	
Other	8315 (8.23%)	65 (8.29%)		1297 (82.7%)	635 (81.0%)	
**Sex:**			0.869			0.693
Male	52462 (51.9%)	410 (52.3%)		835 (53.3%)	410 (52.3%)	
Female	48550 (48.1%)	374 (47.7%)		733 (46.7%)	374 (47.7%)	
**Marital.status:**			0.023			0.886
Married	57136 (56.6%)	489 (62.4%)		971 (61.9%)	489 (62.4%)	
Divorced/Separated	11020 (10.9%)	77 (9.82%)		149 (9.50%)	77 (9.82%)	
UnMarried	13672 (13.5%)	95 (12.1%)		178 (11.4%)	95 (12.1%)	
Widowed	15362 (15.2%)	101 (12.9%)		225 (14.3%)	101 (12.9%)	
Unknown	3822 (3.78%)	22 (2.81%)		45 (2.87%)	22 (2.81%)	
**Grade**			<0.001.			0.889
I	3965 (3.93%)	3 (0.38%)		7 (0.45%)	3 (0.38%)	
II	16685 (16.5%)	100 (12.8%)		177 (11.3%)	100 (12.8%)	
III	15773 (15.6%)	299 (38.1%)		605 (38.6%)	299 (38.1%)	
IV	574 (0.57%)	14 (1.79%)		28 (1.79%)	14 (1.79%)	
Unknown	64015 (63.4%)	368 (46.9%)		751 (47.9%)	368 (46.9%)	
**Primary.Site:**			<0.001			0.94
Body	12721 (12.6%)	112 (14.3%)		217 (13.8%)	112 (14.3%)	
Head	52949 (52.4%)	350 (44.6%)		712 (45.4%)	350 (44.6%)	
Tail	13122 (13.0%)	176 (22.4%)		360 (23.0%)	176 (22.4%)	
Other	22220 (22.0%)	146 (18.6%)		279 (17.8%)	146 (18.6%)	
**Seer.stage:**			0.003			0.866
Localized	7714 (7.64%)	64 (8.16%)		131 (8.35%)	64 (8.16%)	
Regional	36100 (35.7%)	323 (41.2%)		628 (40.1%)	323 (41.2%)	
Distan	57198 (56.6%)	397 (50.6%)		809 (51.6%)	397 (50.6%)	
**AJCC.stage:**			<0.001			0.675
I	5869 (5.81%)	50 (6.38%)		109 (6.95%)	50 (6.38%)	
II	22602 (22.4%)	247 (31.5%)		474 (30.2%)	247 (31.5%)	
III	8931 (8.84%)	59 (7.53%)		102 (6.51%)	59 (7.53%)	
IV	45102 (44.7%)	297 (37.9%)		633 (40.4%)	297 (37.9%)	
Unknown	18508 (18.3%)	131 (16.7%)		250 (15.9%)	131 (16.7%)	
**T.stage**			<0.001			0.904
T1	2841 (2.81%)	5 (0.64%)		9 (0.57%)	5 (0.64%)	
T2	18140 (18.0%)	138 (17.6%)		302 (19.3%)	138 (17.6%)	
T3	33363 (33.0%)	350 (44.6%)		681 (43.4%)	350 (44.6%)	
T4	17124 (17.0%)	106 (13.5%)		210 (13.4%)	106 (13.5%)	
Unknown	29544 (29.2%)	185 (23.6%)		366 (23.3%)	185 (23.6%)	
**LN metastasis**			<0.001			0.411
Yes	42022 (41.6%)	311 (39.7%)		599 (38.2%)	311 (39.7%)	
No	29767 (29.5%)	282 (36.0%)		608 (38.8%)	282 (36.0%)	
Unknown	29223 (28.9%)	191 (24.4%)		361 (23.0%)	191 (24.4%)	
**Distant metastasis**			<0.001			0.508
No	39017 (38.6%)	365 (46.6%)		702 (44.8%)	365 (46.6%)	
Yes	45073 (44.6%)	297 (37.9%)		633 (40.4%)	297 (37.9%)	
Unknow	16922 (16.8%)	122 (15.6%)		233 (14.9%)	122 (15.6%)	
**Surgery:**			<0.001			0.846
No	79832 (79.0%)	475 (60.6%)		958 (61.1%)	475 (60.6%)	
Yes	21180 (21.0%)	309 (39.4%)		610 (38.9%)	309 (39.4%)	
**Radiation:**			0.835			0.074
No	82878 (82.0%)	646 (82.4%)		1338 (85.3%)	646 (82.4%)	
Yes	18134 (18.0%)	138 (17.6%)		230 (14.7%)	138 (17.6%)	
**Chemotherapy:**			0.212			0.988
No	43921 (43.5%)	323 (41.2%)		644 (41.1%)	323 (41.2%)	
Yes	57091 (56.5%)	461 (58.8%)		924 (58.9%)	461 (58.8%)	

### Survival Analysis

**Figure 3:**
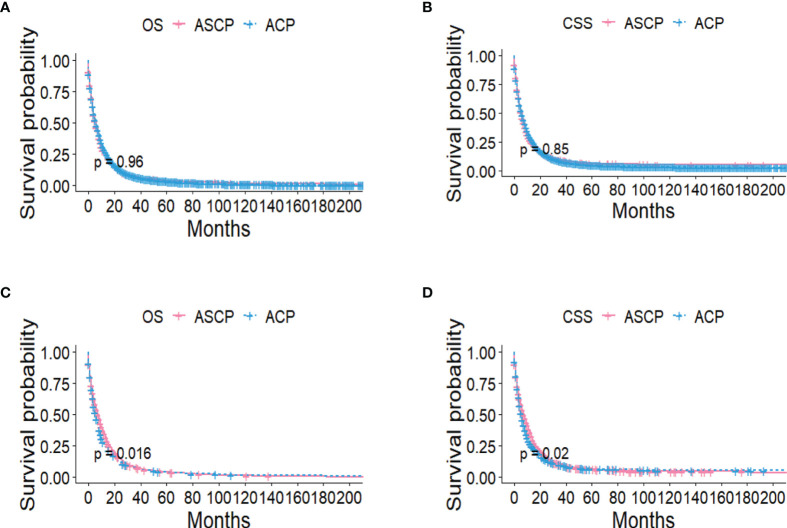
OS and CSS of ACP and ASCP before PSM **(A, B)** and after PSM **(C, D)** (PSM, propensity score matching).

**Figure 4:**
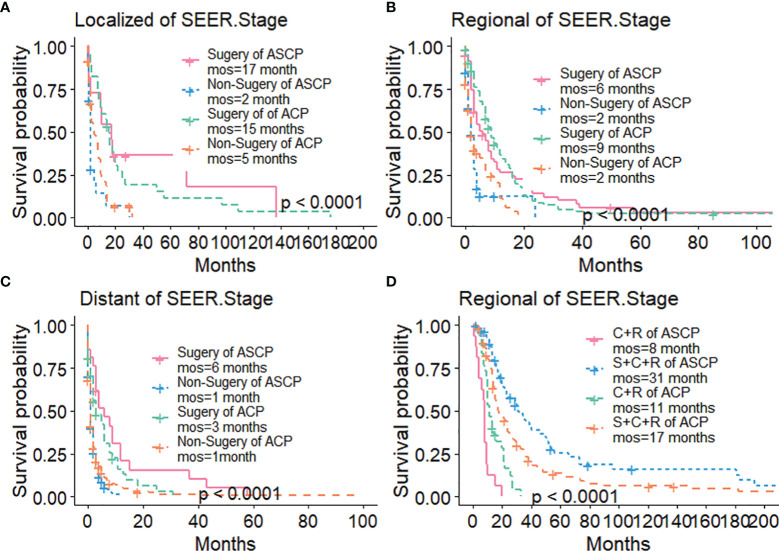
Survival Curves of ACP and ASCP: **(A)** Surgical Treatment in Localized Stage; **(B)** Surgical Treatment in Regional Stage; **(C)** Surgical Treatment in Distant Stage; **(D)** Comparison of Treatment Methods in Regional Stage (S, surgery; C, chemotherapy; R, radiotherapy).

**Figure 5:**
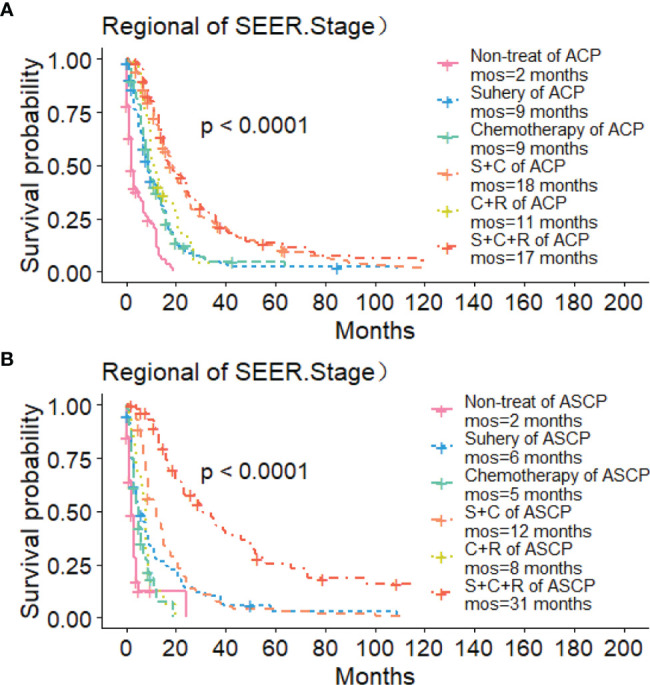
Role of Radiotherapy in ACP and ASCP: **(A)** Role of Radiotherapy in ACP Regional Stage; **(B)** Role of Radiotherapy in ASCP Regional Stage (S, surgery; C, chemotherapy; R, radiotherapy).

**Figure 6:**
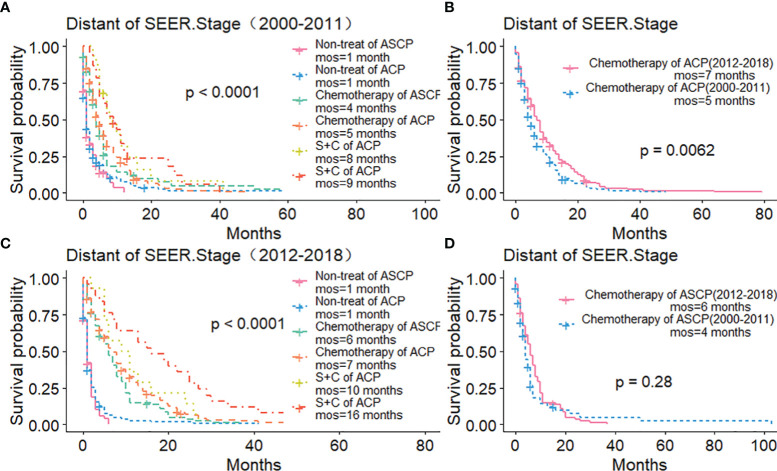
Role of Chemotherapy in ACP and ASCP (2000–2011 and 2012–2018): **(A, C)** Comparison of Treatment Methods between ACP and ASCP in Distant Stage; **(B, D)** Comparison of Chemotherapy Efficacy between ACP and ASCP in Different Time Periods in Distant Stage (S, surgery; C, chemotherapy).

The Kaplan-Meier method was used primarily to evaluate OS and CSS before and after PSM in all patients of both types. Before matching (**Figures 3A, B**), the OS and CSS of ASCP patients were lower than those of ACP patients, but there was no significant difference between the two groups (P = 0.96, P = 0.85). After matching (**Figures 3C, D**), the OS and CSS of ASCP patients were still lower than those of ACP patients, with statistical significance (P = 0.016 and P = 0.02 respectively),ASCP and ACP patients had a median OS of 6 months and 7 months respectively. In addition, the matched data was used to compare the OS of ASCP and ACP patients without any treatment and after surgery, radiotherapy and chemotherapy. In the two groups of patients who underwent surgery, considering that they were at different stages of tumor and their prognoses were also different, we plotted the survival curves of three stages according to SEER.Stage. At all stages (**Figures 4A–C**), ACP or ASCP patients who underwent surgery had a better prognosis than those who did not. Notably, the post-operative MOS of ASCP patients were higher than those of ACP patients in both the Localized and Distant stages, but were lower than those of ACP patients in the Regional stage (6 months vs 9 months) (P < 0.001). A total of 368 subjects were treated with adjuvant radiotherapy in this study, of which 249 were treated in the Regional stage, often in combination with adjuvant chemotherapy. In patients with ACP (**Figure 5A**), the MOS of patients receiving surgery+chemotherapy were higher than those receiving surgery+adjuvant chemotherapy+adjuvant radiotherapy (18 months vs. 17 months) (P < 0.001). Surprisingly, (**Figure 5B**) the MOS were significantly higher in ASCP patients receiving concurrent surgery+adjuvant radiotherapy+adjuvant chemotherapy than in those receiving other treatment modalities, reaching a staggering 31 months (P < 0.001). In addition, as shown in **Figure 4D**, among the patients receiving surgery+chemotherapy+radiotherapy, the benefits for ASCP patients were significantly higher than those for ACP patients (31 months vs. 17 months) (P < 0.001). It can be seen that ACP patients did not benefit from radiotherapy. Surgery+adjuvant chemotherapy+adjuvant radiotherapy may be the optimal treatment for ASCP patients in the Regional stage. Patients in the Distant stage accounted for 43.62% of all patients. According to the 2011 literature, pancreatic cancer is treated by a multi-drug combination regimen. Due to monotherapy, taking 2011 as the boundary, we divided the patients into two groups to assess the role of chemotherapy at this stage (**Figures 6A, C**). From 2000 to 2011, among ASCP and ACP patients, MOS were 4 months and 5 months respectively. From 2012 to 2018, the two showed improved MOS of 6 and 7 months respectively, and this difference was statistically significant in ACP patients (P = 0.0062) (**Figure 6B**), whereas it was not significant in ASCP patients (P = 0.28) (**Figure 6D**).

### Cox Regression Analysis

According to the inclusion and exclusion criteria, 784 patients were included in the ASCP group, randomly divided into the training group (N = 552) and test group (N = 232) in a ratio of 7:3. **Supplementary Table 3** lists the basic demographic and clinical characteristics, with no significant differences between the two groups. As shown in **Table 2**, Cox univariate analysis suggested that age, marital status, race, SEER.Stage, AJCC.Stage, lymph node metastasis, distant metastasis, surgery (yes/no), adjuvant radiotherapy (yes/no), adjuvant chemotherapy (yes/no) and tumor size were independent prognostic factors that ultimately affected patient survival; while Cox multivariate analysis suggested that age, race, SEER.Stage, AJCC.Stage, surgery (yes/no), radiotherapy (yes/no), chemotherapy (yes/no) and tumor size were independent prognostic factors that ultimately affected patient survival. Cox multivariate analysis excluded lymph node metastasis and M stage, which are considered to have a strong correlation with AJCC.Stage.

**Table 2:** Univariate and multivariate cox regression analyses of the prognoses of the ASCP.

Variables	Univariate analysis				Multivariate analysis		
	HR	95%CI	P		HR	95%CI	P
**Age**(years)	1.019	1.01~1.029	<0.001		1.019	1.008~1.03	0.001
**Marital status**							
Married	Reference						
Divorced/Separated	1.018	0.754~1.374	0.908		1.007	0.738~1.375	0.963
Unmarried	1.455	1.109~1.909	0.007		1.323	0.999~1.752	0.051
Widowed	1.474	1.13~1.922	0.004		1.151	0.757~1.343	0.955
Unknow	1.498	0.891~2.519	0.127		1.008	0.664`1.994	0.617
**Race**							
White	Reference						
Black	1.344	1.022~1.768	0.035		1.58	1.191~2.096	0.002
Other	0.935	0.665~1.316	0.70		1.047	0.739~1.485	0.794
**Sex**							
Male	Reference						
Female	1.082	0.907~1.292	0.383				
**Primary site**	Reference						
Body							
Head	0.842	0.643~1.102	0.21				
Tail	0.907	0.669~1.229	0.527				
Other	1.175	0.863~1.599	0.306				
**Grade**							
I	Reference						
II	1.098	0.268~4.502	0.896				
III	1.333	0.33~5.38	0.686				
IV	1.131	0.228~5.612	0.88				
Unknow	2.457	0.61~9.901	0.206				
**Seer.stage**							
Localized	Reference						
Regional	0.389	0.27~0.561	<0.001		0.82	0.422~1.594	0.559
Distant	0.39	0.321~0.475	<0.001		0.699	0.502~0.974	0.035
**AJCC.stage**							
I	Reference						
II	1.246	0.788	1.97	0.347	2.469	1.111~5.487	0.026
III	1.844	1.072	3.172	0.027	2.223	0.965~5.122	0.061
IV	3.181	2.021	5.006	<0.001	2.034	0.774~5.345	0.15
Unknown	1.963	1.219	3.162	0.006	1.443	0.46~4.53	0.53
**T stage**							
T1	Reference						
T2	1.8	0.568~5.704	0.318				
T3	1.638	0.524~5.124	0.396				
T4	2.491	0.785~7.907	0.121				
Unknow	2.347	0.746~7.389	0.145				
**Lymph node metastasis**							
No	Reference						
Yes vs	0.988	0.805~1.212	0.907		1.007	0.806~1.258	0.954
Unknow vs No	1.279	1.015~1.61	0.037		1.047	0.752~1.459	0.785
**Distant metastasis**							
No	Reference						
Yes	2.38	1.944~2.914	<0.001		1.008	0.599~1.696	0.977
Unknow	1.427	1.108~1.837	0.006		1.361	0.45~4.117	0.586
**Surgery**							
No	Reference						
Yes	0.297	0.242~0.364	<0.001		0.354	0.267~0.469	<0.001
**Radiotherapy**							
No	Reference						
Yes	0.452	0.352~0.58	<0.001		0.617	0.469~0.813	0.001
**Chemotherapy**					0.479	0.39~0.588	<0.001
No	Reference						
Yes	0.461	0.384~0.553	<0.001		1.437	1.15~1.796	0.001
**Tumor size (cm)**							
<4.6	Reference						
4.7~7.0	1.62	1.309~2.004	<0.001		1.437	1.15~1.796	0.001
>7.0 vs <4.6	2.475	1.876~3.266	<0.001		1.984	1.477~2.664	<0.001
Unknow vs <4.6	2.544	1.925~3.362	<0.001		1.368	1.009~1.855	0.043

### Construction and Validation of Nomogram

**Figure 7:**
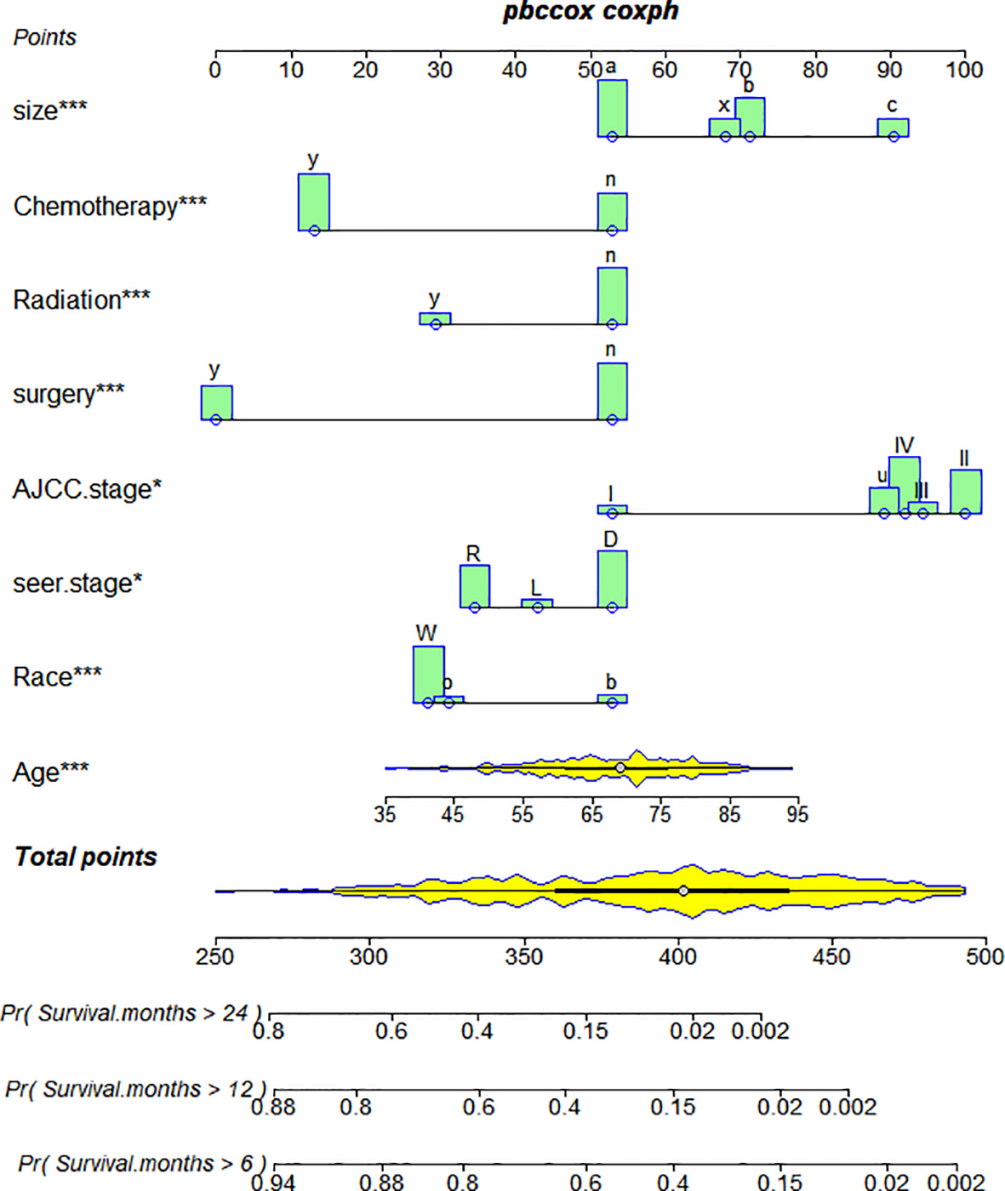
Nomogram of ASCP. *P<0.05 for independent prognostic factors screened in the multivariate cox regression analysis of ASCP prognosis. ***P<0.01 of independent prognostic factors screened in the multivariate cox regression analysis of ASCP prognosis. They can reflect the influence of the independent prognostic factor on the prognosis of patients, and "***" is more strongly correlated with the prognosis of patients than "*".

**Figure 8:**
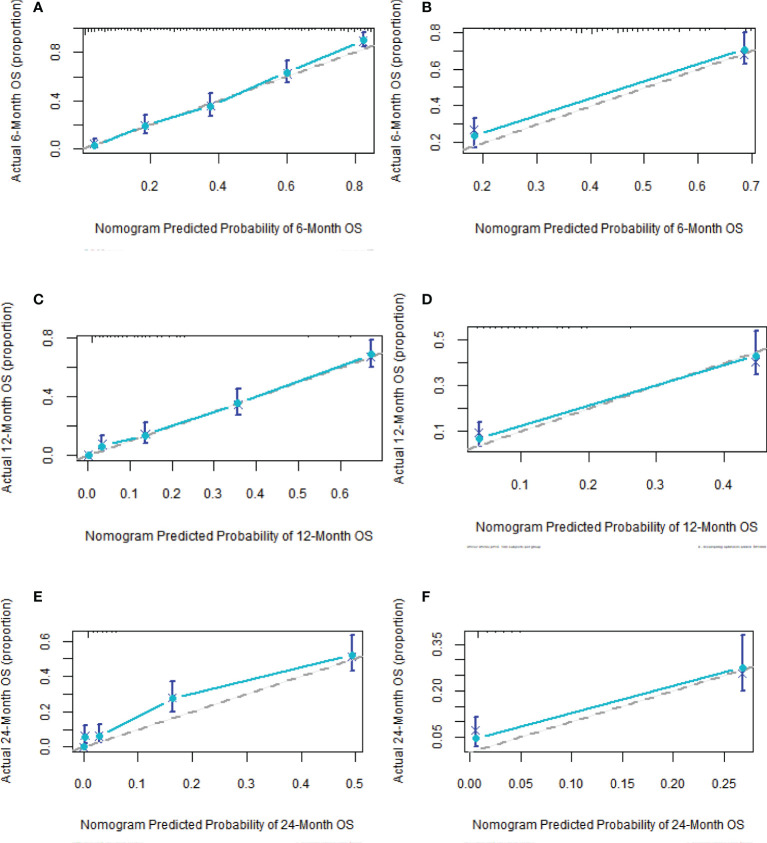
Calibration curves of nomogram **(A, C, F)** in training group at 6, 12 and 24 months; calibration curves of nomogram **(B, D, E)** in validation group at 6, 12 and 24 months.

**Figure 9:**
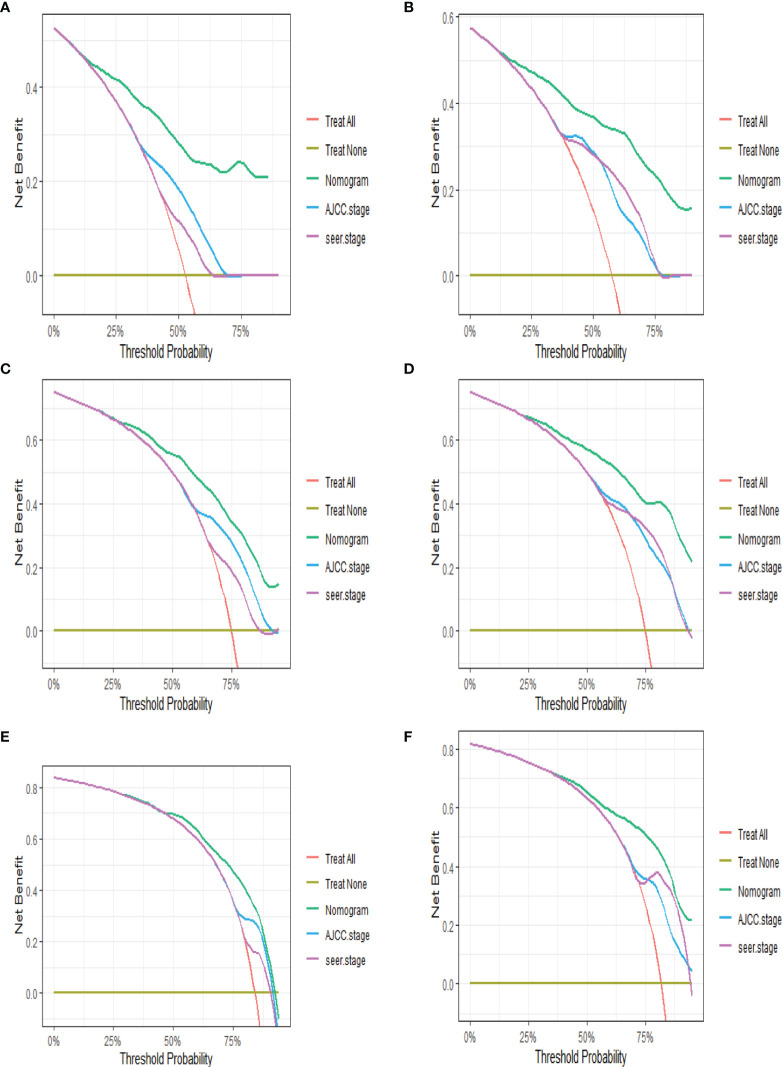
DCA of nomogram **(A, C, F)** in training group at 6, 12 and 24 months; DCA of nomogram **(B, D, E)** in validation group at 6, 12 and 24 months (DCA, decision curve analysis).

Based on Cox analysis, 8 independent prognostic factors were used to construct the nomogram (**Figure 7**), and a specific score for each independent prognostic factor was obtained (**Supplementary Table 4**). According to the total score, the survival of ASCP patients at 6, 12 and 24 months can be confidently predicted. The stability of the nomogram was verified by the correction curves in the training and verification groups. **Figure 8** shows that there is good consistency between the predicted survival results and actual survival results. **Figure 9** demonstrates the superiority of this model over AJCC.Stage and SEER.Stage in predicting the net clinical benefits of 6, 12 and 24-month survival.

### Comparison of OS Prediction Accuracy Between Nomogram, AJCC.Stage and SEER.Stage

**Figure 10:**
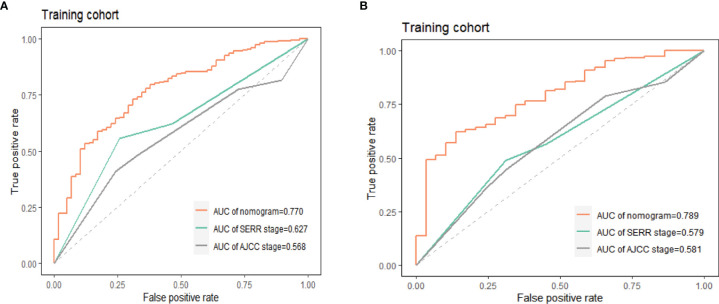
Accuracy comparison of nomogram, AJCC.Stage, SEER.Stage in training group **(A)** and validation group **(B)**, AUC, area under the curve; ROC, receiver operating characteristic.

The accuracy of the prediction model was verified by the area under the ROC curve. As shown in **Figure 10**, in the training group, the AUCs of the nomogram, AJCC.Stage and SEER.Stage were 0.770, 0.567 and 0.626 respectively; in the training group, the AUCs of the nomogram, AJCC.Stage and SEER.Stage were 0.789, 0.579 and 0.581 respectively. The results show that this model has higher accuracy in predicting the OS of ASCP patients, making it more suitable for predicting the survival prognosis of ASCP.

## Discussion

In this population-based retrospective analysis, the incidence of ASCP is extremely low, and the overall incidence of pancreatic cancer is about 0.7%, which is similar to previous studies (4, 23). It is worth noting that the incidence of ASCP and ACP is increasing year by year from 2000 to 2018, which may be related to pancreatic inflammation and obesity (24). This study is the first to use PSM to compare demographic characteristics between ASCP and ACP. The results show that ASCP and ACP are very similar in age of onset, gender and race; the average age of onset is 67, and the incidence is highest in white males, this has been reported in previous studies (14, 16, 25). In terms of clinicopathological characteristics, it is worth noting that Grade II in pathology has a lower OS than Grade IV, which may be related to data sources and caused by bias; more prospective studies may be needed for validation. Interestingly, consistent with previous research (15, 25, 26), the histological grading of ASCP indicates a higher degree of malignancy, with Grades III and IV accounting for about 40%, compared with only about 16% for ACP, this may be related to the squamous cell component (27). In addition, the two have similar clinical manifestations, such as abdominal pain, weight loss, and jaundice, making it difficult to distinguish ASCP from ACP.Therefore, endoscopic ultrasound-guided fine-needle aspiration (EUS-FNA) is extremely important for the preoperative diagnosis of solid pancreatic lesions and is considered the gold standard (28–30).

Many studies (15, 31) have shown that ASCP is always more aggressive and malignant histologically than ACP, but previous studies on ASCP (10, 14, 16) have confirmed that there is no difference in OS between ASCP and ACP in the whole population, which is different from the conclusions reported in the literature (8, 12, 25). In order to arrive at a reliable conclusion and minimize the influence of various biases, we used PSM to balance the baseline characteristics. It was found that before PSM, there was no difference in OS and CSS between ASCP and ACP, but after PSM, ACP had a good prognosis with a median OS of 7 months, while the median survival time for ASCP was 6 months (P < 0.05). This conclusion is based on the fact that the data sample is by far the largest, so the conclusion is highly reliable. Subsequently, we compared the prognosis improvement of ASCP and ACP by different treatment methods.Surgery remains a vital treatment modality for pancreatic cancer and may significantly prolong the OS of patients (5, 13, 32–34). According to the results of this study, the surgical rate of ASCP patients is significantly higher than that of ACP patients (up to nearly 40%), and both ASCP and ACP patients can benefit from surgery. Pancreatic cancer can be divided into three categories: resectable, borderline and unresectable (35). However, since this is not documented in the SEER database, we demonstrate from the three stages of SEER.Stage that patients can benefit from surgery when it is available. Studies (36, 37) have shown that the FOLFIRINOX regimen and the gemcitabine plus albumin-bound paclitaxel regimen have significantly improved the prognosis of metastatic pancreatic cancer. Besides, in the updated NCCN guidelines for pancreatic cancer, the multi-drug combination therapy model is adopted as the acceptable regimen for advanced pancreatic cancer (38). In this study, it is also demonstrated that the prognosis of ACP patients receiving chemotherapy after 2012 in the Distant stage was significantly higher than before (7 months vs. 5 months) (P < 0.05), and the survival time after ASCP chemotherapy was increased as compared with before 2011 (6 months vs. 4 months), but not statistically significant. Our results suggest that in the Regional stage, the survival time of ASCP and ACP patients undergoing chemotherapy after surgery is significantly higher than that of patients undergoing surgery alone, which is consistent with the literature (39, 40). Unfortunately, chemotherapy regimens are not recorded in the SEER database, which is one of the limitations of this study. Besides, radiotherapy plays a vital role in the treatment of pancreatic adenosquamous carcinoma. Our results suggest that in the Regional stage, the MOS of ASCP reaches 31 months after surgery combined with adjuvant radiotherapy and chemotherapy, which is significantly higher than that of ACP (17 months). This indicates that radiotherapy can significantly improve the overall survival time of ASCP patients (41–43), and surgery+adjuvant radiotherapy+adjuvant chemotherapy fails to significantly improve the prognosis of ACP patients, the reason might be that squamous carcinoma in ASCP is more sensitive to radiotherapy (43), Compared with ACP, the role of non-surgical treatment in patients with ASCP is unclear (14), and in practice, due to limited data on ASCP, standard chemotherapy regimens for ACP such as FOLFIRINOX, gemcitabine and capecitabine may be used (44, 45), so we need to further explore the standard treatment of ASCP. It is noteworthy that recent studies (46) have shown that the expression of PD-L1 is limited to squamous cell components, which may be the key to subsequent targeted therapies. The role of squamous cells in ASC also requires further investigation. In addition, neoadjuvant therapy may play an important role in the future to improve the R0 resection rate in patients with locally advanced or distant metastases (47–49).

In order to further explore the independent prognostic risk factors for ASCP, we performed univariate and multivariate Cox analysis, which revealed that the prognosis of advanced-stage white elderly patients was poor, while surgery, radiotherapy and chemotherapy were protective factors (50–53)that were beneficial for prognosis. This is consistent with the previous survival analysis. According to the multivariate Cox analysis, we established a nomogram with C-indexes of 0.780 and 0.781 respectively, indicating good consistency between predicted survival rate and actual survival rate. We then validated the prediction effects of the nomogram at 6, 12 and 24 months, which revealed that the nomogram has good performance. DCA indicated that the net clinical benefit of this model is higher than that of AJCC.Stage and SEER.Stage. Finally, the area under the ROC curve indicated that the prediction of this model is more comprehensive and accurate. This model may be used for individualized prognostic assessment, and may become an effective diagnostic tool for making treatment-related decisions (21).

However, our analysis had several limitations. First, since it is a retrospective analysis, the selection bias was inevitable even if PSM was used to reduce the bias. Second, the absence of smoking and drinking history, pancreatitis history and CA19-9 and CA125 data in the SEER database may have affected the screening of independent prognostic factors. Third, since the SEER database fails to record the primary type of tumor before surgery such as resectable, borderline and unresectable for surgical treatment, this imposes certain limitations on the analysis of this study. Fourth, this study covers a period of 18 years during which the replacement of the chemotherapy regimen, monotherapy to multi-drug combination and the emergence of neoadjuvant therapy were included. However, in this study, we only included adjuvant therapy (radiotherapy and chemotherapy), but not neoadjuvant therapy; if neoadjuvant therapy had been included, there would have been a lot of missing data, imposing a limitation on this study. Fifth, the lack of external data does not support external validation, so this prediction model may not be highly applicable to populations in other regions. To this end, ASCP patients at our center will be collected for external validation at a later date.

## Conclusion

In conclusion, the incidence of both ACP and ASCP is increasing year by year, which requires greater attention. in the Regional stage, surgery + chemotherapy + radiotherapy can significantly improve the prognosis of ASCP. Finally, the accuracy of our prediction model is higher than that of AJCC.Stage and SEER.Stage, and can help clinicians to better implement individual treatment.

## Data Availability Statement

The original contributions presented in the study are included in the article/**Supplementary Material**. Further inquiries can be directed to the corresponding author.

## Author Contributions

S-YL designed the study and wrote the manuscript. Z-QY and M-JL extracted the data and conducted statistical analysis. S-YL and C-NX made and improved the charts. Z-MW controlled the quality of the study and revised the paper. All authors contributed to the paper and approved the submission of the manuscript.

## Conflict of Interest

The authors declare that the research was conducted in the absence of any commercial or financial relationships that could be construed as a potential conflict of interest.

## Publisher’s Note

All claims expressed in this article are solely those of the authors and do not necessarily represent those of their affiliated organizations, or those of the publisher, the editors and the reviewers. Any product that may be evaluated in this article, or claim that may be made by its manufacturer, is not guaranteed or endorsed by the publisher.

## References

[1] Siegel RL Miller KD Jemal A. Cancer Statistics, 2018. CA Cancer J Clin (2018) 68(1):7–30. doi: 10.3322/caac.21442 29313949

[2] Khalaf N El-Serag HB Abrams HR Thrift AP. Burden of Pancreatic Cancer: From Epidemiology to Practice. Clin Gastroenterol Hepatol (2021) 19(5):876–84. doi: 10.1016/j.cgh.2020.02.054 PMC8559554 32147593

[3] Klein AP. Pancreatic Cancer Epidemiology: Understanding the Role of Lifestyle and Inherited Risk Factors. Nat Rev Gastroenterol Hepatol (2021) 18(7):493–502. doi: 10.1038/s41575-021-00457-x 34002083 PMC9265847

[4] Fitzgerald TL Hickner ZJ Schmitz M Kort EJ. Changing Incidence of Pancreatic Neoplasms: A 16-Year Review of Statewide Tumor Registry. Pancreas (2008) 37(2):134–8. doi: 10.1097/MPA.0b013e318163a329 18665072

[5] Okabayashi T Hanazaki K. Surgical Outcome of Adenosquamous Carcinoma of the Pancreas. World J Gastroenterol (2008) 14(44):6765–70. doi: 10.3748/wjg.14.6765 PMC2773870 19058301

[6] Kardon DE Thompson LD Przygodzki RM Heffess CS. Adenosquamous Carcinoma of the Pancreas: A Clinicopathologic Series of 25 Cases. Mod Pathol (2001) 14(5):443–51. doi: 10.1038/modpathol.3880332 11353055

[7] Makiyama K Takuma K Zea-Iriarte WL Ikuno N Kawatomi M Mori N. Adenosquamous Carcinoma of the Pancreas. J Gastroenterol (1995) 30(6):798–802. doi: 10.1007/BF02349652 8963403

[8] Kaiser J Hinz U Mayer P Hank T Niesen W Hackert T. Clinical Presentation and Prognosis of Adenosquamous Carcinoma of the Pancreas - Matched-Pair Analysis With Pancreatic Ductal Adenocarcinoma. Eur J Surg Oncol (2021) 47(7):1734–41. doi: 10.1016/j.ejso.2021.02.011 33622577

[9] Hsu JT Yeh CN Chen YR Chen HM Hwang TL Jan YY. Adenosquamous Carcinoma of the Pancreas. Digestion (2005) 72(2-3):104–8. doi: 10.1159/000088364 16172546

[10] Hester CA Augustine MM Choti MA Mansour JC Minter RM Polanco PM. Comparative Outcomes of Adenosquamous Carcinoma of the Pancreas: An Analysis of the National Cancer Database. J Surg Oncol (2018) 118(1):21–30. doi: 10.1002/jso.25112 29878370

[11] Borazanci E Millis SZ Korn R Han H Whatcott CJ Gatalica Z. Adenosquamous Carcinoma of the Pancreas: Molecular Characterization of 23 Patients Along With a Literature Review. World J Gastrointest Oncol (2015) 7(9):132–40. doi: 10.4251/wjgo.v7.i9.132 PMC4569590 26380056

[12] Boecker J Feyerabend B Tiemann K Buchwalow I Wagner KC Oldhafer KJ. Adenosquamous Carcinoma of the Pancreas Comprise a Heterogeneous Group of Tumors With the Worst Outcome: A Clinicopathological Analysis of 25 Cases Identified in 562 Pancreatic Carcinomas Resected With Curative Intent. Pancreas (2020) 49(5):683–91. doi: 10.1097/MPA.0000000000001548 32433407

[13] Moslim MA Lefton MD Ross EA Mackrides N Reddy SS. Clinical and Histological Basis of Adenosquamous Carcinoma of the Pancreas: A 30-Year Experience. J Surg Res (2021) 259:350–6. doi: 10.1016/j.jss.2020.09.024 PMC8902409 33190924

[14] Katz MH Taylor TH Al-Refaie WB Hanna MH Imagawa DK Anton-Culver H. Adenosquamous Versus Adenocarcinoma of the Pancreas: A Population-Based Outcomes Analysis. J Gastrointest Surg (2011) 15(1):165–74. doi: 10.1007/s11605-010-1378-5 PMC3023036 21082275

[15] Lenkiewicz E Malasi S Hogenson TL Flores LF Barham W Phillips WJ. Genomic and Epigenomic Landscaping Defines New Therapeutic Targets for Adenosquamous Carcinoma of the Pancreas. Cancer Res (2020) 80(20):4324–34. doi: 10.1158/0008-5472.CAN-20-0078 PMC7906529 32928922

[16] Boyd CA Benarroch-Gampel J Sheffield KM Cooksley CD Riall TS. 415 Patients With Adenosquamous Carcinoma of the Pancreas: A Population-Based Analysis of Prognosis and Survival. J Surg Res (2012) 174(1):12–9. doi: 10.1016/j.jss.2011.06.015 PMC3210865 21816433

[17] Kamarajah SK Burns WR Frankel TL Cho CS Nathan H. Validation of the American Joint Commission on Cancer (AJCC) 8th Edition Staging System for Patients With Pancreatic Adenocarcinoma: A Surveillance, Epidemiology and End Results (SEER) Analysis. Ann Surg Oncol (2017) 24(7):2023–30. doi: 10.1245/s10434-017-5810-x 28213792

[18] Sheng W Dong M Wang G Shi X Gao W Wang K. The Diversity Between Curatively Resected Pancreatic Head and Body-Tail Cancers Based on the 8th Edition of AJCC Staging System: A Multicenter Cohort Study. BMC Cancer (2019) 19(1):981. doi: 10.1186/s12885-019-6178-z 31640615 PMC6805668

[19] Hue JJ Katayama E Sugumar K Winter JM Ammori JB Rothermel LD. The Importance of Multimodal Therapy in the Management of Nonmetastatic Adenosquamous Carcinoma of the Pancreas: Analysis of Treatment Sequence and Strategy. Surgery (2021) 169(5):1102–9. doi: 10.1016/j.surg.2020.11.026 33376004

[20] Song Z Wang Y Zhou Y Zhang D. A Novel Predictive Tool for Determining the Risk of Early Death From Stage IV Endometrial Carcinoma: A Large Cohort Study. Front Oncol (2020) 10:620240. doi: 10.3389/fonc.2020.620240 33381462 PMC7769006

[21] Balachandran VP Gonen M Smith JJ DeMatteo RP. Nomograms in Oncology: More Than Meets the Eye. Lancet Oncol (2015) 16(4):e173–80. doi: 10.1016/S1470-2045(14)71116-7 PMC4465353 25846097

[22] Park SY. Nomogram: An Analogue Tool to Deliver Digital Knowledge. J Thorac Cardiovasc Surg (2018) 155(4):1793. doi: 10.1016/j.jtcvs.2017.12.107 29370910

[23] Simone CG Zuluaga Toro T Chan E Feely MM Trevino JG George TJ Jr. Characteristics and Outcomes of Adenosquamous Carcinoma of the Pancreas. Gastrointest Cancer Res (2013) 6(3):75–9. doi: 10.1200/jco.2013.31.4_suppl.311 PMC3737509 23936547

[24] Saluja A Maitra A. Pancreatitis and Pancreatic Cancer. Gastroenterology (2019) 156(7):1937–40. doi: 10.1053/j.gastro.2019.03.050 30940522

[25] Imaoka H Shimizu Y Mizuno N Hara K Hijioka S Tajika M. Clinical Characteristics of Adenosquamous Carcinoma of the Pancreas: A Matched Case-Control Study. Pancreas (2014) 43(2):287–90. doi: 10.1097/MPA.0000000000000089 24518509

[26] Liu C Karam R Zhou Y Su F Ji Y Li G. The UPF1 RNA Surveillance Gene is Commonly Mutated in Pancreatic Adenosquamous Carcinoma. Nat Med (2014) 20(6):596–8. doi: 10.1038/nm.3548 PMC4048332 24859531

[27] Hoshimoto S Hoshi N Hishinuma S Shirakawa H Tomikawa M Ozawa I. Clinical Implications of the Proliferative Ability of the Squamous Component Regarding Tumor Progression of Adenosquamous Carcinoma of the Pancreas: A Preliminary Report. Pancreatology (2017) 17(5):788–94. doi: 10.1016/j.pan.2017.08.001 28784574

[28] Crinò SF Larghi A Bernardoni L Parisi A Frulloni L Gabbrielli A. Touch Imprint Cytology on Endoscopic Ultrasound Fine-Needle Biopsy Provides Comparable Sample Quality and Diagnostic Yield to Standard Endoscopic Ultrasound Fine-Needle Aspiration Specimens in the Evaluation of Solid Pancreatic Lesions. Cytopathology (2019) 30(2):179–86. doi: 10.1111/cyt.12662 30484917

[29] Malak M Masuda D Ogura T Imoto A Abdelaal UM Sabet EA. Yield of Endoscopic Ultrasound-Guided Fine Needle Aspiration and Endoscopic Retrograde Cholangiopancreatography for Solid Pancreatic Neoplasms. Scand J Gastroenterol (2016) 51(3):360–7. doi: 10.3109/00365521.2015.1086019 26365063

[30] Yoshinaga S Suzuki H Oda I Saito Y. Role of Endoscopic Ultrasound-Guided Fine Needle Aspiration (EUS-FNA) for Diagnosis of Solid Pancreatic Masses. Dig Endosc (2011) 23 Suppl 1:29–33. doi: 10.1111/j.1443-1661.2011.01112.x 21535197

[31] Zhao X Li H Lyu S Zhai J Ji Z Zhang Z. Single-Cell Transcriptomics Reveals Heterogeneous Progression and EGFR Activation in Pancreatic Adenosquamous Carcinoma. Int J Biol Sci (2021) 17(10):2590–605. doi: 10.7150/ijbs.58886 PMC8315026 34326696

[32] Yamaue H Tanimura H Onishi H Tani M Kinoshita H Kawai M. Adenosquamous Carcinoma of the Pancreas: Successful Treatment With Extended Radical Surgery, Intraoperative Radiation Therapy, and Locoregional Chemotherapy. Int J Pancreatol (2001) 29(1):53–8. doi: 10.1385/IJGC:29:1:53 11558633

[33] Smoot RL Zhang L Sebo TJ Que FG. Adenosquamous Carcinoma of the Pancreas: A Single-Institution Experience Comparing Resection and Palliative Care. J Am Coll Surg (2008) 207(3):368–70. doi: 10.1016/j.jamcollsurg.2008.03.027 18722942

[34] Ito T Sugiura T Okamura Y Yamamoto Y Ashida R Ohgi K. Long-Term Outcomes After an Aggressive Resection of Adenosquamous Carcinoma of the Pancreas. Surg Today (2019) 49(10):809–19. doi: 10.1007/s00595-019-01807-8 30980180

[35] Tempero MA Malafa MP Al-Hawary M Behrman SW Benson AB Cardin DB. Pancreatic Adenocarcinoma, Version 2.2021, NCCN Clinical Practice Guidelines in Oncology. J Natl Compr Canc Netw (2021) 19(4):439–57. doi: 10.6004/jnccn.2021.0017 33845462

[36] Conroy T Desseigne F Ychou M Bouché O Guimbaud R Bécouarn Y. FOLFIRINOX Versus Gemcitabine for Metastatic Pancreatic Cancer. N Engl J Med (2011) 364(19):1817–25. doi: 10.1056/NEJMoa1011923 21561347

[37] Von Hoff DD Ramanathan RK Borad MJ Laheru DA Smith LS Wood TE. Gemcitabine Plus Nab-Paclitaxel is an Active Regimen in Patients With Advanced Pancreatic Cancer: A Phase I/II Trial. J Clin Oncol (2011) 29(34):4548–54. doi: 10.1200/JCO.2011.36.5742 PMC3565012 21969517

[38] Tempero MA Malafa MP Behrman SW Benson AB 3rd Casper ES Chiorean EG. Pancreatic Adenocarcinoma, Version 2.2014: Featured Updates to the NCCN Guidelines. J Natl Compr Canc Netw (2014) 12(8):1083–93. doi: 10.6004/jnccn.2014.0106 25099441

[39] Connell CM Brais R Whitaker H Upponi S Beh I Risdall J. Early Relapse on Adjuvant Gemcitabine Associated With an Exceptional Response to 2nd Line Capecitabine Chemotherapy in a Patient With Pancreatic Adenosquamous Carcinoma With Strong Intra-Tumoural Expression of Cytidine Deaminase: A Case Report. BMC Cancer (2020) 20(1):38. doi: 10.1186/s12885-020-6516-1 31941506 PMC6964020

[40] Fang Y Pu N Zhang L Wu W Lou W. Chemoradiotherapy is Associated With Improved Survival for Resected Pancreatic Adenosquamous Carcinoma: A Retrospective Cohort Study From the SEER Database. Ann Transl Med (2019) 7(20):522. doi: 10.21037/atm.2019.10.12 31807504 PMC6861782

[41] Shibagaki K Fujita K Nakayama S Takenaka M Fukuba N Matsui S. Complete Response of a Pancreatic Adenosquamous Carcinoma to Chemoradiotherapy. Int J Clin Oncol (2008) 13(1):74–7. doi: 10.1007/s10147-007-0690-x 18307024

[42] Cardenes HR Moore AM Johnson CS Yu M Helft P Chiorean EG. A Phase II Study of Gemcitabine in Combination With Radiation Therapy in Patients With Localized, Unresectable, Pancreatic Cancer: A Hoosier Oncology Group Study. Am J Clin Oncol (2011) 34(5):460–5. doi: 10.1097/COC.0b013e3181e9c103 20881474

[43] Gruhl JD Garrido-Laguna I Francis SR Affolter K Tao R Lloyd S. The Impact of Squamous Cell Carcinoma Histology on Outcomes in Nonmetastatic Pancreatic Cancer. Cancer Med (2020) 9(5):1703–11. doi: 10.1002/cam4.2851 PMC7050091 31945808

[44] Neoptolemos JP Palmer DH Ghaneh P Psarelli EE Valle JW Halloran CM. Comparison of Adjuvant Gemcitabine and Capecitabine With Gemcitabine Monotherapy in Patients With Resected Pancreatic Cancer (ESPAC-4): A Multicentre, Open-Label, Randomised, Phase 3 Trial. Lancet (2017) 389(10073):1011–24. doi: 10.1016/S0140-6736(16)32409-6 28129987

[45] Chin V Nagrial A Sjoquist K O'Connor CA Chantrill L Biankin AV. Chemotherapy and Radiotherapy for Advanced Pancreatic Cancer. Cochrane Database Syst Rev (2018) 3(3):Cd011044. doi: 10.1002/14651858.CD011044.pub2 29557103 PMC6494171

[46] Tanigawa M Naito Y Akiba J Kawahara A Okabe Y Ishida Y. PD-L1 Expression in Pancreatic Adenosquamous Carcinoma: PD-L1 Expression is Limited to the Squamous Component. Pathol Res Pract (2018) 214(12):2069–74. doi: 10.1016/j.prp.2018.10.006 30477643

[47] Nitsche U Wenzel P Siveke JT Braren R Holzapfel K Schlitter AM. Resectability After First-Line FOLFIRINOX in Initially Unresectable Locally Advanced Pancreatic Cancer: A Single-Center Experience. Ann Surg Oncol (2015) 22(Suppl 3):S1212–20. doi: 10.1245/s10434-015-4851-2 26350368

[48] Ferrone CR Marchegiani G Hong TS Ryan DP Deshpande V McDonnell EI. Radiological and Surgical Implications of Neoadjuvant Treatment With FOLFIRINOX for Locally Advanced and Borderline Resectable Pancreatic Cancer. Ann Surg (2015) 261(1):12–7. doi: 10.1097/SLA.0000000000000867 PMC4349683 25599322

[49] Petrelli F Coinu A Borgonovo K Cabiddu M Ghilardi M Lonati V. FOLFIRINOX-Based Neoadjuvant Therapy in Borderline Resectable or Unresectable Pancreatic Cancer: A Meta-Analytical Review of Published Studies. Pancreas (2015) 44(4):515–21. doi: 10.1097/MPA.0000000000000314 25872127

[50] Voong KR Davison J Pawlik TM Uy MO Hsu CC Winter J. Resected Pancreatic Adenosquamous Carcinoma: Clinicopathologic Review and Evaluation of Adjuvant Chemotherapy and Radiation in 38 Patients. Hum Pathol (2010) 41(1):113–22. doi: 10.1016/j.humpath.2009.07.012 PMC3556992 19801164

[51] Wild AT Dholakia AS Fan KY Kumar R Moningi S Rosati LM. Efficacy of Platinum Chemotherapy Agents in the Adjuvant Setting for Adenosquamous Carcinoma of the Pancreas. J Gastrointest Oncol (2015) 6(2):115–25. doi: 10.3978/j.issn.2078-6891.2014.091. PMC4311088 25830031

[52] Brunetti O Aprile G Marchetti P Vasile E Casadei Gardini A Scartozzi M. Systemic Chemotherapy for Advanced Rare Pancreatic Histotype Tumors: A Retrospective Multicenter Analysis. Pancreas (2018) 47(6):759–71. doi: 10.1097/MPA.0000000000001063 29771769

[53] Ishii H Furuse J Boku N Okusaka T Ikeda M Ohkawa S. Phase II Study of Gemcitabine Chemotherapy Alone for Locally Advanced Pancreatic Carcinoma: JCOG0506. Jpn J Clin Oncol (2010) 40(6):573–9. doi: 10.1093/jjco/hyq011 20185458

