# Supplementary figures and images for: Survival Analysis and Prediction Model of ASCP Based on SEER Database
Source: Front Oncol. 2022 Jun 24;12:909257. doi: 10.3389/fonc.2022.909257 (PMC9263703; doi:10.3389/fonc.2022.909257)

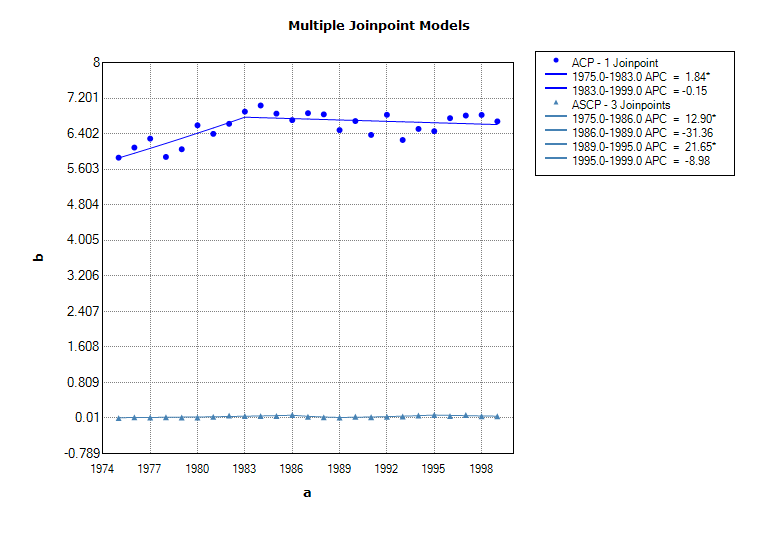

Supplement: Supplementary file 1 [file Image_1.tiff]
