# Supplementary material for: Survival Analysis and Prediction Model of ASCP Based on SEER Database
Source: Front Oncol. 2022 Jun 24;12:909257. doi: 10.3389/fonc.2022.909257 (PMC9263703; doi:10.3389/fonc.2022.909257)
Supplement: Supplementary file 2 [file Table_1.docx]

Age-adjusted incidence rates of ASCP and ACP from 2000 to 2018（Supplementary Table 1）

| Histology | Year of diagnosis | Age-Adjusted Rate/Trend | Standard Error | Lower Confidence Interval | Upper Confidence Interval |
| --- | --- | --- | --- | --- | --- |
| ACP |  | 29.321 |  |  |  |
| ACP |  | 1.433 |  | 1.27 | 1.596 |
| ACP | 2000 | 5.294 | 0.085 | 5.128 | 5.465 |
| ACP | 2001 | 5.252 | 0.085 | 5.088 | 5.42 |
| ACP | 2002 | 5.408 | 0.085 | 5.243 | 5.577 |
| ACP | 2003 | 5.363 | 0.084 | 5.2 | 5.53 |
| ACP | 2004 | 5.621 | 0.085 | 5.455 | 5.79 |
| ACP | 2005 | 5.785 | 0.086 | 5.619 | 5.956 |
| ACP | 2006 | 5.808 | 0.085 | 5.641 | 5.978 |
| ACP | 2007 | 5.917 | 0.085 | 5.751 | 6.087 |
| ACP | 2008 | 6.212 | 0.087 | 6.044 | 6.384 |
| ACP | 2009 | 6.257 | 0.086 | 6.089 | 6.428 |
| ACP | 2010 | 6.119 | 0.084 | 5.955 | 6.287 |
| ACP | 2011 | 6.267 | 0.084 | 6.102 | 6.434 |
| ACP | 2012 | 6.23 | 0.083 | 6.069 | 6.395 |
| ACP | 2013 | 6.44 | 0.083 | 6.278 | 6.606 |
| ACP | 2014 | 6.477 | 0.083 | 6.316 | 6.641 |
| ACP | 2015 | 6.59 | 0.082 | 6.429 | 6.753 |
| ACP | 2016 | 6.622 | 0.082 | 6.463 | 6.784 |
| ACP | 2017 | 6.646 | 0.081 | 6.488 | 6.806 |
| ACP | 2018 | 6.847 | 0.081 | 6.689 | 7.008 |
| ASCP |  | 123.35 |  |  |  |
| ASCP |  | 3.672 |  | 1.896 | 5.479 |
| ASCP | 2000 | 0.026 | 0.006 | 0.016 | 0.041 |
| ASCP | 2001 | 0.052 | 0.008 | 0.037 | 0.071 |
| ASCP | 2002 | 0.044 | 0.008 | 0.03 | 0.062 |
| ASCP | 2003 | 0.026 | 0.006 | 0.016 | 0.04 |
| ASCP | 2004 | 0.028 | 0.006 | 0.018 | 0.042 |
| ASCP | 2005 | 0.04 | 0.007 | 0.027 | 0.056 |
| ASCP | 2006 | 0.03 | 0.006 | 0.019 | 0.045 |
| ASCP | 2007 | 0.038 | 0.007 | 0.026 | 0.055 |
| ASCP | 2008 | 0.037 | 0.007 | 0.025 | 0.053 |
| ASCP | 2009 | 0.043 | 0.007 | 0.03 | 0.06 |
| ASCP | 2010 | 0.036 | 0.006 | 0.024 | 0.051 |
| ASCP | 2011 | 0.059 | 0.008 | 0.044 | 0.077 |
| ASCP | 2012 | 0.06 | 0.008 | 0.045 | 0.078 |
| ASCP | 2013 | 0.049 | 0.007 | 0.036 | 0.066 |
| ASCP | 2014 | 0.052 | 0.008 | 0.038 | 0.069 |
| ASCP | 2015 | 0.067 | 0.008 | 0.052 | 0.086 |
| ASCP | 2016 | 0.057 | 0.008 | 0.043 | 0.074 |
| ASCP | 2017 | 0.061 | 0.008 | 0.047 | 0.078 |
| ASCP | 2018 | 0.059 | 0.007 | 0.045 | 0.075 |
