# Supplementary material for: Survival Analysis and Prediction Model of ASCP Based on SEER Database
Source: Front Oncol. 2022 Jun 24;12:909257. doi: 10.3389/fonc.2022.909257 (PMC9263703; doi:10.3389/fonc.2022.909257)
Supplement: Supplementary file 3 [file Table_2.docx]

Age-adjusted incidence rates of ASCP and ACP from 1975 to 1999（Supplementary Table 2）

| Histology | Year of diagnosis | Age-Adjusted Rate/Trend | Standard Error | Lower Confidence Interval | Upper Confidence Interval |
| --- | --- | --- | --- | --- | --- |
| ACP | 1975 | 58.68 | 1.9 | 55.02 | 62.52 |
| ACP | 1976 | 60.95 | 1.92 | 57.25 | 64.83 |
| ACP | 1977 | 62.96 | 1.94 | 59.22 | 66.87 |
| ACP | 1978 | 58.84 | 1.84 | 55.28 | 62.56 |
| ACP | 1979 | 60.57 | 1.86 | 56.97 | 64.33 |
| ACP | 1980 | 65.96 | 1.93 | 62.22 | 69.85 |
| ACP | 1981 | 64.03 | 1.88 | 60.39 | 67.83 |
| ACP | 1982 | 66.29 | 1.9 | 62.61 | 70.11 |
| ACP | 1983 | 69.04 | 1.92 | 65.33 | 72.91 |
| ACP | 1984 | 70.41 | 1.93 | 66.68 | 74.3 |
| ACP | 1985 | 68.58 | 1.89 | 64.93 | 72.38 |
| ACP | 1986 | 67.12 | 1.84 | 63.55 | 70.83 |
| ACP | 1987 | 68.69 | 1.85 | 65.1 | 72.42 |
| ACP | 1988 | 68.42 | 1.83 | 64.87 | 72.1 |
| ACP | 1989 | 64.88 | 1.76 | 61.47 | 68.43 |
| ACP | 1990 | 66.92 | 1.79 | 63.46 | 70.5 |
| ACP | 1991 | 63.79 | 1.72 | 60.46 | 67.25 |
| ACP | 1992 | 68.32 | 1.76 | 64.91 | 71.86 |
| ACP | 1993 | 62.66 | 1.67 | 59.43 | 66.02 |
| ACP | 1994 | 65.14 | 1.69 | 61.86 | 68.53 |
| ACP | 1995 | 64.64 | 1.67 | 61.41 | 67.99 |
| ACP | 1996 | 67.56 | 1.69 | 64.28 | 70.96 |
| ACP | 1997 | 68.15 | 1.69 | 64.88 | 71.53 |
| ACP | 1998 | 68.29 | 1.67 | 65.05 | 71.65 |
| ACP | 1999 | 66.84 | 1.64 | 63.66 | 70.14 |
| ASCP | 1975 | 0.12 | 0.09 | 0.01 | 0.43 |
| ASCP | 1976 | 0.28 | 0.15 | 0.07 | 0.71 |
| ASCP | 1977 | 0.26 | 0.11 | 0.08 | 0.6 |
| ASCP | 1978 | 0.3 | 0.12 | 0.11 | 0.66 |
| ASCP | 1979 | 0.24 | 0.12 | 0.06 | 0.6 |
| ASCP | 1980 | 0.25 | 0.12 | 0.08 | 0.6 |
| ASCP | 1981 | 0.39 | 0.15 | 0.15 | 0.8 |
| ASCP | 1982 | 0.68 | 0.19 | 0.36 | 1.16 |
| ASCP | 1983 | 0.56 | 0.17 | 0.28 | 1.01 |
| ASCP | 1984 | 0.55 | 0.17 | 0.27 | 0.99 |
| ASCP | 1985 | 0.59 | 0.17 | 0.3 | 1.04 |
| ASCP | 1986 | 0.82 | 0.2 | 0.48 | 1.32 |
| ASCP | 1987 | 0.41 | 0.14 | 0.18 | 0.78 |
| ASCP | 1988 | 0.28 | 0.12 | 0.1 | 0.62 |
| ASCP | 1989 | 0.25 | 0.12 | 0.08 | 0.59 |
| ASCP | 1990 | 0.43 | 0.15 | 0.2 | 0.82 |
| ASCP | 1991 | 0.32 | 0.12 | 0.13 | 0.66 |
| ASCP | 1992 | 0.41 | 0.14 | 0.19 | 0.77 |
| ASCP | 1993 | 0.47 | 0.15 | 0.22 | 0.86 |
| ASCP | 1994 | 0.7 | 0.17 | 0.4 | 1.13 |
| ASCP | 1995 | 0.82 | 0.19 | 0.49 | 1.28 |
| ASCP | 1996 | 0.55 | 0.15 | 0.29 | 0.94 |
| ASCP | 1997 | 0.82 | 0.18 | 0.5 | 1.26 |
| ASCP | 1998 | 0.53 | 0.15 | 0.28 | 0.9 |
| ASCP | 1999 | 0.53 | 0.15 | 0.28 | 0.9 |
