# Supplementary material for: Survival Analysis and Prediction Model of ASCP Based on SEER Database
Source: Front Oncol. 2022 Jun 24;12:909257. doi: 10.3389/fonc.2022.909257 (PMC9263703; doi:10.3389/fonc.2022.909257)
Supplement: Supplementary file 4 [file Table_3.docx]

**Supplementary Table3**|Demographic and clinicopathological characteristics of the included population

| **Variables** | **Total**  **n=784(%)** | **Training**  **n=552(%)** | **Validation n=232(%)** | **P** |
| --- | --- | --- | --- | --- |
| **Age (years)** | 69 (35~95) | 69 (35~94) | 69 (35~95) | 0.915 |
| **Marital status** |  |  |  | 0.788 |
| Married | 489 (62.4%) | 337(61.10%) | 152(65.50%) |  |
| Divorce | 77 (9.8%) | 55 (10.0%) | 22 (9.5%) |  |
| Unmarried | 95 (12.1%) | 70 (12.7%) | 25 (10.8%) |  |
| Unknow | 22 (2.8%) | 17 (3.1%) | 5 (2.2%) |  |
| Widowed | 101 (12.9%) | 73 (13.2%) | 28 (12.1%) |  |
| **Race** |  |  |  | 0.703 |
| White | 635 (81.0%) | 446 (80.8%) | 189 (81.5%) |  |
| Black | 84 (10.70%) | 62 (11.20%) | 22 (9.50%) |  |
| Other | 65 (8.3%) | 44 (8.0%) | 21 (9.1%) |  |
| **Sex** |  |  |  |  |
| Female | 374 (47.7%) | 265 (48.0%) | 109 (47.0%) |  |
| Male | 410 (52.3%) | 287 (52.0%) | 123 (53.0%) |  |
| **Primary site** |  |  |  | 0.829 |
| BOP | 112 (14.3%) | 79 (14.3%) | 33 (14.2%) |  |
| HOP | 350 (44.6%) | 250 (45.3%) | 100 (43.1%) |  |
| Other | 146 (18.6%) | 104 (18.8%) | 42 (18.1%) |  |
| TOP | 176 (22.4%) | 119 (21.6%) | 57 (24.6%) |  |
| **Grade** |  |  |  |  |
| I | 3 (0.4%) | 2 (0.4%) | 1 (0.4%) | 0.207 |
| II | 100 (12.8%) | 72 (13.0%) | 28 (12.1%) |  |
| III | 299 (38.1%) | 207 (37.5%) | 92 (39.7%) |  |
| IV | 14 (1.8%) | 6 (1.1%) | 8 (3.4%) |  |
| Unknow | 368 (46.9%) | 265 (48.0%) | 103 (44.4%) |  |
| **Seer stage** |  |  |  | 0.300 |
| Localized | 64 (8.2%) | 45 (8.2%) | 19 (8.2%) |  |
| Regional | 323 (41.2%) | 218 (39.5%) | 105 (45.3%) |  |
| Distant | 397 (50.6%) | 289 (52.4%) | 108 (46.6%) |  |
| **AJCC stage** |  |  |  | 0.325 |
| I | 50 (6.4%) | 31 (5.6%) | 19 (8.2%) |  |
| II | 247 (31.5%) | 167 (30.3%) | 80 (34.5%) |  |
| III | 59 (7.5%) | 40 (7.2%) | 19 (8.2%) |  |
| IV | 297 (37.9%) | 217 (39.3%) | 80 (34.5%) |  |
| Unknow | 131 (16.7%) | 97 (17.6%) | 34 (14.7%) |  |
| **T stage** |  |  |  | 0.330 |
| T1 | 5 (0.6%) | 5 (0.9%) | 0 (0.0%) |  |
| T2 | 138 (17.6%) | 95 (17.2%) | 43 (18.5%) |  |
| T3 | 350 (44.6%) | 238 (43.1%) | 112 (48.3%) |  |
| T4 | 106 (13.5%) | 78 (14.1%) | 28 (12.1%) |  |
| Unknow | 185 (23.6%) | 136 (24.6%) | 49 (21.1%) |  |
| **Lymph node metastasis** |  |  |  | 0.357 |
| No | 311 (39.7%) | 210 (38.0%) | 101 (43.5%) |  |
| Yes | 282 (36.0%) | 204 (37.0%) | 78 (33.6%) |  |
| Unknow | 191 (24.4%) | 138 (25.0%) | 53 (22.8%) |  |
| **Distant metastasis** |  |  |  | 0.537 |
| No | 394 (50.3%) | 261 (47.3%) | 133 (57.3%) |  |
| Yes | 268 (34.2%) | 200 (36.2%) | 68 (29.3%) |  |
| Unknow | 122 (15.6%) | 91 (16.5%) | 31 (13.4%) |  |
| **Surgery** |  |  |  | 0.091 |
| No | 475 (60.6%) | 345 (62.5%) | 130 (56.0%) |  |
| Yes | 309 (39.4%) | 207 (37.5%) | 102 (44.0%) |  |
| **Radiotherapy** |  |  |  | 0.657 |
| No | 646 (82.4%) | 457 (82.8%) | 189 (81.5%) |  |
| Yes | 138 (17.6%) | 95 (17.2%) | 43 (18.5%) |  |
| **Chemotherapy** |  |  |  | 0.238 |
| No | 323 (41.2%) | 220 (39.9%) | 103 (44.4%) |  |
| Yes | 461 (58.8%) | 332 (60.1%) | 129 (55.6%) |  |
| **Tumor size (cm)** |  |  |  | 0.946 |
| <4.6 | 343 (43.8%) | 241 (43.7%) | 102 (44.0%) |  |
| 4.7~7.0 | 239 (30.5%) | 166 (30.1%) | 73 (31.5%) |  |
| >7.0 | 103 (13.1%) | 73 (13.2%) | 30 (12.9%) |  |
| Unknow | 99 (12.6%) | 72 (13.0%) | 27 (11.6%) |  |
