# Supplementary material for: Survival Analysis and Prediction Model of ASCP Based on SEER Database
Source: Front Oncol. 2022 Jun 24;12:909257. doi: 10.3389/fonc.2022.909257 (PMC9263703; doi:10.3389/fonc.2022.909257)
Supplement: Supplementary file 5 [file Table_4.docx]

| **Variables** | **Scores** |
| --- | --- |
| **Age (years)** | 0.9* Age-8.5 |
| **Race** |  |
| White | 28 |
| Black | 53 |
| Other | 31 |
| **Seer stage** |  |
| Localized | 43 |
| Regional | 34 |
| Distant | 53 |
| **AJCC stage** |  |
| I | 53 |
| II | 100 |
| III | 94 |
| IV | 92 |
| Unknow | 89 |
| **Surgery** |  |
| Yes | 0 |
| No | 53 |
| **Radiotherapy** |  |
| Yes | 29 |
| No | 53 |
| **Chemotherapy** |  |
| Yes | 13 |
| No | 53 |
| **Tumor size (cm)** |  |
| <4.6 | 53 |
| 4.7~7.0 | 71 |
| >7.0 | 90 |
| Unknow | 68 |

**Supplementary Table4**|Scores for Each Independent Risk Factor in Nomograms
